# Beliefs about social dynamics and open science

**DOI:** 10.1098/rsos.230061

**Published:** 2025-05-21

**Authors:** Ashley Thomas, Chris Bourg, Rebecca Saxe

**Affiliations:** ^1^ Psychology Department, Harvard University, Cambridge, MA, USA; ^2^ Libraries, Massachusetts Institute of Technology, Cambridge, MA, USA; ^3^ Department of Brain and Cognitive Sciences, Massachusetts Institute of Technology, Cambridge, MA, USA

**Keywords:** beliefs, social, dynamics, science, meta-science, social dynamics

## Abstract

Open science advocates argue that publicly and freely available scientific manuscripts, data and code will have wide-reaching collective benefits. However, the adoption of open science practices may depend on the fit between researchers' perceptions of open science and the social dynamics of their field. For example, if researchers understand open science as primarily a means of cooperating with other researchers, its adoption may be faster and more effective among researchers who see their field as less competitive and less hierarchical. The present studies operationalize open science attitudes as plans to publicly share manuscripts/preprints, code, stimuli/instruments and data, as well as participants’ perceptions of the importance of these practices. In Study 1, researchers perceived the social dynamics of their field (competition and hierarchy) as distinct from the traits of individuals in their field (warmth and competence). In Study 2, neither researchers’ perceptions of social dynamics, nor their view of open science as motivated by cooperation, predicted their attitudes to open science practices. However, attitudes about open science were generally very positive among researchers who opt-in to a study about open science, limiting the variance to be explained. Moreover, people’s self-reported motivations for sharing manuscripts and materials differed from their perceptions of why others share manuscripts and materials. Study 3 tested the same questions in an independent and more representative sample. Results of Study 3 agreed with results of Study 2: neither researchers’ perceptions of social dynamics, nor their view of open science as motivated by cooperation, predicted their open science practices. Again, attitudes about open science were generally very positive among researchers even in this representative sample and people’s self-reported motivations for sharing manuscripts and materials differed from their perceptions of why others share manuscripts and materials.

## 1. Introduction

Advocates of open science argue that sharing scientific products (e.g. manuscripts, stimuli, protocols, data, etc.) could improve science by making findings more verifiable and replicable, making scientific progress more efficient and making scientific findings more accessible. While these practices are increasingly mandated by funding agencies [1] and institutions [2], both the effectiveness and longevity of these mandates will depend on the way that these practices are adopted. Open science poses a collective action problem: even though most people agree open science practices could improve science, it is not always in each individual’s narrow self-interest to engage in these practices [3]. For example, someone may fulfil a mandate to share data but do so in a way that is useless to other scholars [4,5]. Moreover, many scholars fail to share data even when mandates are in place [6–8].

Several studies have asked about the obstacles that prevent people from engaging in open science (refer to [9] for a widely used survey tool). Both in surveys and in interviews, researchers often say that engaging in open science, such as sharing data and materials, would be useful for scientific progress [7]. When they are asked why they do not engage in these practices, researchers say it is because they have insufficient time or resources, lack training, want to be the first to publish findings from their data and/or have concerns about potential misuse of their data [7,10–14]. When asked why they do share, researchers say they are motivated to foster transparency and reproducibility or to allow other researchers to validate their findings [11].

Since key obstacles to open science include missing resources and training, making open science practices easier and less time-intensive should increase their use. New infrastructure, such as free data repositories and preprint servers, have led to much wider adoption of open science practices [15–17]. But providing infrastructure is only the first step, and the maintenance of this infrastructure depends on people’s engagement with it. Complementary to improving infrastructure, researchers will be more likely to engage in open science practices if such practices are perceived as common and standard in their discipline [16,18]. Changing the perception of descriptive norms is integral to the ‘Pyramid of Culture Change’ [19] and EAST (Easy, Attractive, Social and Timely; [20]) models. Advocates of these models recommend that open science practices be made visible in talks and on websites, to increase the perceived frequency of these behaviours in the research community.

While these strategies likely have affected change in the research community, increasing the perceived frequency of open science practices alone is unlikely to change researchers’ behaviour. In other contexts, behaviour change is not simply a product of the perceived frequency of other people’s behaviours [21]. Rather, target behaviours must be portrayed and perceived as valued by a relevant community (refer to [22] for a discussion about the social dynamics of scientific communities and [23] for a discussion of social dynamics and open science practices). Thus, in the context of scientific research, open science practices may be most likely to be adopted by researchers who perceive those practices as aligned with the values and rules for advancement in their scientific community.

Here, we test whether researchers’ plans to implement open science are predicted by their perceptions of the social dynamics in their field and their ideas about why they and others engage in open science. We operationalize open science attitudes as plans to publicly share manuscripts/preprints, code, stimuli/instruments and data, as well as participants’ perceptions of the importance of these practices. We operationalize the perception of social dynamics as the extent to which people see their field as hierarchical and competitive. Finally, we operationalize people’s perception of motivations for open science by asking participants the extent to which they and others are motivated to engage in open science as a way to cooperate with other researchers, compared to other motives like fulfilling mandates or as a way to gain prestige.

We hypothesize that people’s plans to share manuscripts and materials will be predicted by the fit between the social dynamics of their field and the motivations for open science. For example, a researcher who sees their field as cooperative and sees open science as a way to cooperate with other researchers may be more likely to engage in open science practices. On the other hand, a researcher who sees their field as hierarchical and competitive and sees open science as a way to gain prestige may be more likely to engage in open science practices.

We begin by asking whether researchers’ perceptions of the social dynamics of their field are coherent. Specifically, we ask whether they perceive social dynamics as distinct from the way they perceive characteristics of people in their field (i.e. how warm and competent people are). We focused on two dimensions with which social groups can vary—how hierarchical/participatory a group is and how cooperative/competitive a group is. Previous research that asks how people think about groups has largely focused on people’s stereotypes of societal groups (e.g. ‘wealthy people’, ’immigrants’). This research has found robust evidence that people tend to think about groups along two dimensions: warmth (do I expect that group to have interests aligned with my own?) and competence (do I expect that group to be able to achieve its interests effectively?) [24]. In other words, this research asks whether people represent common traits or characteristics of people who belong to social groups. People outside of academia may stereotype researchers as cold and competent [25,26]. However, researchers themselves must learn something else about their group: To become an effective member of a social group, people must learn about the group’s social structure, social dynamics or its ‘rules of engagement’ [27,28]. When researchers join their academic field, they need to recognize the degree to which their group is hierarchical and competitive, or participatory and cooperative. These social processes may be distinct from warmth and competence since they apply to properties of interpersonal interactions between individuals rather than to the traits of individuals. Therefore, in Study 1, we created and validated a measure of researchers’ perceptions of social dynamics in their field.

Then, in Studies 2 and 3, we ask whether researchers’ perceptions of social dynamics, along with their perception of motivations for open science, predict their open science attitudes. These two studies differ only in the sampling strategy. A key limitation in carrying out research on open science attitudes is sampling bias. Most of the studies cited above (e.g. [7,10–14]) recruited samples via email, social media or listservs, or by randomly selecting authors of journal articles. Participation rates in these ‘opt-in’ studies are often low (for surveys that described their response rate it was typically below 5%). People who have strong beliefs or positive attitudes about open science may be more likely to participate. Like other surveys on open science, we used convenience samples in Studies 1 (advertising our survey on Twitter and listservs) and 2 (sending the survey out to all researchers in a school of science at a major research university) and achieved low to moderate participation rates. In contrast, in Study 3, we collected a more representative sample of researcher opinions by distributing the survey at the beginning of regularly scheduled laboratory meetings in laboratory groups evenly sampled from five scientific departments at a major research university. Using this approach, the participation rate was high (95% of people who attended the meetings took the survey).

In sum, the overall goal of these three studies was to test whether researchers’ perceptions of the social dynamics in their field interact with their perception of the motivations for open science to predict which researchers will openly share their materials and manuscripts.

## 2. Study 1

The purpose of Study 1 was to establish whether researchers represent the social dynamics of their field (hierarchy and competitiveness) distinctly from the traits (warmth and competence) of people in their field. We pre-registered (https://osf.io/zwjc3) the following hypotheses: H1: We hypothesized that the perceptions researchers have of their field will be characterized by two dimensions: (i) decision-making is hierarchical vs participatory and (ii) researchers in the field compete versus cooperate. An alternative hypothesis, H2, was that most of the variance in researchers’ perceptions can be captured by a single dimension, corresponding to positive versus negative evaluations. The null hypothesis, H3, was that the variation in researchers’ perceptions of their fields reduces to previously established dimensions of social perception of individuals and groups: warmth and competence. Support for H3 would mean that we failed to measure perceptions of social dynamics, distinct from stereotypes of group members.

### 2.1. Study 1 methods

#### 2.1.1. Study 1 participants

**Figure 1.**
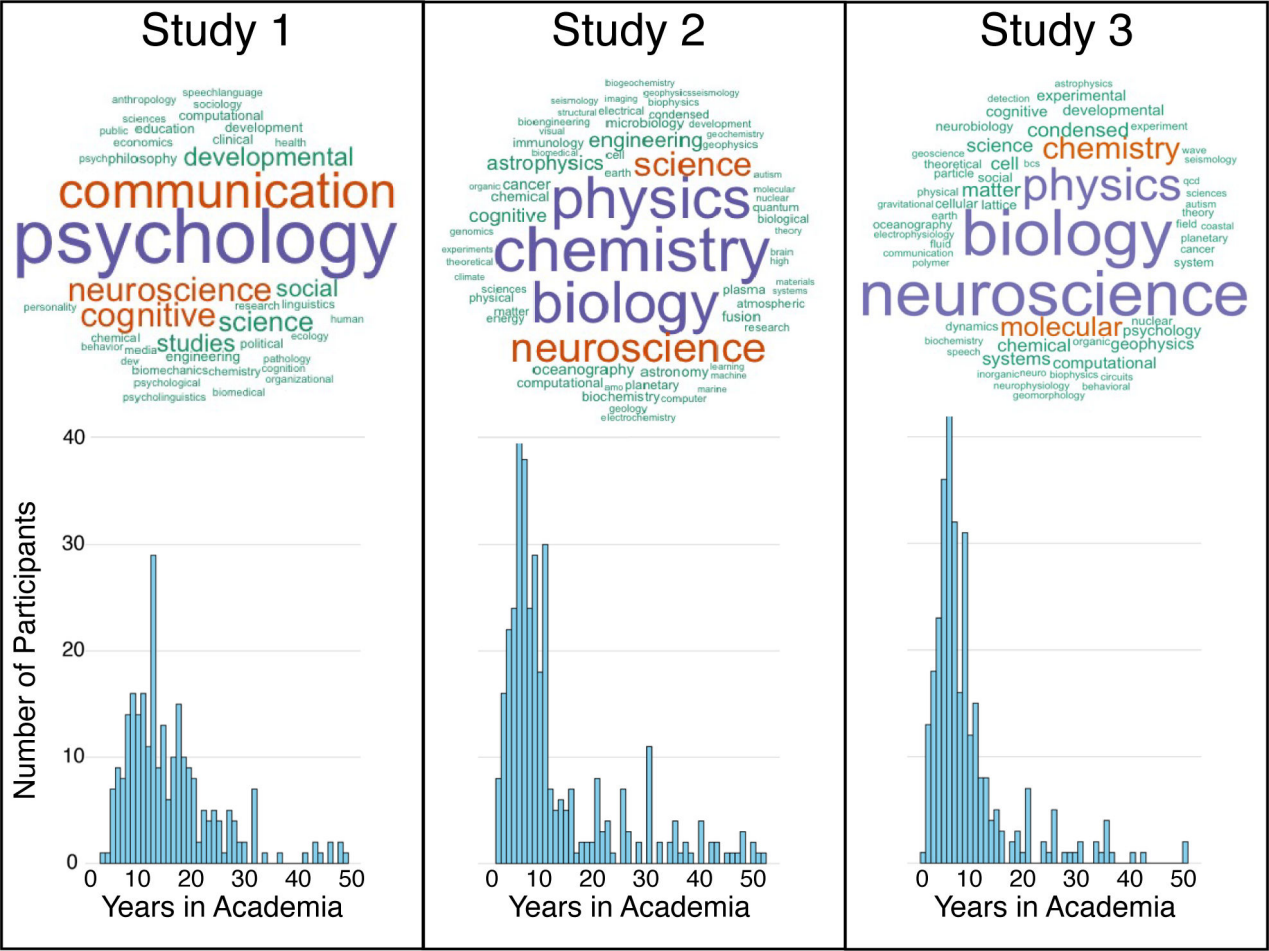
*Top row*: Word clouds based on frequency of response to, ‘What is your Field?’ for each study. *Bottom row*: Histograms of the number of years participants have been in academia.

Participants were recruited via the personal Twitter accounts of all three authors and field-specific listservs (Cog Sci, Cognitive Development Society and Communications), where we announced we were conducting a survey about open science open to all researchers. *n* = 315 researchers participated in our survey. Because these are potentially sensitive topics, we only collected three pieces of demographic information to protect anonymity: the number of years that the participants had been in their field, the extent that the participant identifies with their field and the participant’s academic field (refer to figure 1 for histograms of the number of years the participants have been in their field, as well as word clouds that describe the frequency with which participants entered words in the free response question asking them, ‘What is your field?’). No participants were excluded from the entire survey. If participants failed to answer a question, they were excluded from the analysis that involved that question. The stopping rule we used and pre-registered was 300 participants or until 1 month had passed after we initially posted the survey. We did not perform a power analysis because we did not feel confident in estimating the effect size for this novel instrument.

#### 2.1.2. Study 1 materials

The survey asked participants about social dynamics and warmth and competence of individuals in their field. The text of questions is included below and the full text of the survey can be found here: https://osf.io/4g2tu/. Refer to electronic supplementary material for more histograms (electronic supplementary material, section 1.1.1), and additional analyses.

To ask about participants' perceptions of social dynamics in their field, we asked participants how strongly they agreed with the following statements:

(1) **Hierarchy**: *There is a hierarchy, ranking or pecking order in (participant field), and the people at the top have the most influence on which scholarship is valued*. (hypothesized: hierarchy factor)
(2) **Junior**: *In (participant field), even graduate student researchers who have exciting new ideas or perspectives are able to influence which scholarship is valued*. (hypothesized: hierarchy factor)
(3) **Norms.Prestige**: *The norms and best practices in (participant field) are decided by the highest-ranking members or most prestigious researchers without much input or influence from less well-known researchers*. (hypothesized: hierarchy factor)
(4) **Norms.Less.Well.Known**. *Less well-known researchers have as much or more influence on the norms and best practices in the field of (participant field) compared to prestigious or well-known researchers*. (hypothesized: hierarchy factor)
(5) **Seminar**: *At a typical symposium or seminar talk in (participant field), graduate students and early career researchers ask as many questions as senior faculty*. (hypothesized: hierarchy factor)
(6) **Conferences**: *In (participant field), questions addressed to graduate students and early career researchers are likely to be patronizing or hostile*. (hypothesized: hierarchy factor)
(7) **Stealing.Ideas**: *In order to succeed in (participant field) researchers must be wary of other researchers who may try to steal ideas or disrupt others’ research*. (hypothesized: cooperation factor)
(8) **Zero.Sum**: *Success in (participant field) is a zero-sum game that inevitably has a few winners and many losers*. (hypothesized: cooperation factor)
(9) **Collaborate**: *The biggest advances and the most original ideas in (participant field) arise from people working together collaboratively*. (hypothesized: cooperation factor)
(10) **Cooperate**: *In order to succeed in (participant field) researchers must cooperate with other researchers*. (hypothesized: cooperation factor)

To ask about warmth and competence, we asked the extent to which participants agreed that other researchers in their field were Friendly, Kind, Likable, Nice, Capable, Competent, Efficient and Skillful.

#### 2.1.3. Study 1 analysis approach

We used a combination of exploratory factor analyses and confirmatory factor analyses. For all factor analyses, we first performed a Kaiser–Meyer–Olkin (KMO) test for Sampling Adequacy to ask whether sampling is adequate and Bartlett’s test for sphericity to ask whether there was redundancy within the variables, such that they might load onto factors. The results of these tests are reported in the electronic supplementary material, sections 1.1.3, 2.14, 2.3.3 and 2.4.3. In Study 1, we used JASP to perform exploratory factor analyses to test whether we had evidence for four factors (warmth, competence, hierarchy and cooperation) or whether the questions about social dynamics loaded onto warmth and competence. We also used the ‘psych’ package in R [29] to calculate BIC values for models with different numbers of factors and used the function ‘bic_to_bf’ to compute Bayes Factors that compare these models. Note that this analysis was not pre-registered, and we performed this analysis after a suggestion from a reviewer. We pre-registered that we would calculate Cronbach’s alpha for each subscale, but subsequently were convinced that Cronbach’s alpha does not measure reliability, so instead we report McDonald’s ω and Guttman’s λ2 [30], using the unidimensional reliability analysis function in JASP. We compared reliability for the items in each subscale to measures for all items. Refer to electronic supplementary materials for extra analyses, including correlation matrices (e.g. electronic supplementary material, section 1.1.4) and analyses about what predicted people’s perceptions of social dynamics (electronic supplementary material, sections 1.2, 2.2 and 3.2).

### 2.2. Study 1 results

#### 2.2.1. Factor and reliability analysis

H1 was partially supported. The questions about social dynamics did not load onto one factor and did not load onto the questions about warmth and competence. However, the factor structure was somewhat different than we had predicted. We used exploratory factor analysis to investigate the structure of all questions. In line with H1, we found evidence for 4 factors (χ^2^ = 158, *p* < 0.001). We also used BIC to compute Bayes Factors, which compared a model with 4 factors to models with 1, 2 and 3 factors (note that this was not pre-registered, and a reviewer suggested it). We found evidence that the data fit a model with 4 factors better than models with 1, 2, 3 or 5 factors (BF for 4 factors compared to 1: BF₁₀ = 1.5 × 10^135^; BF₁₀ for 4 factors compared to 2: BF₁₀ = 1.0 × 10^37^; BF₁₀ for 4 factors compared to 3: BF₁₀ = 15.2, BF₁₀ for 4 factors compared to 5: BF₁₀ = 2.7 × 10^10^). Ruling out H3, the questions about social dynamics did not load onto the same factors as the traits of individuals.

**Figure 2.**
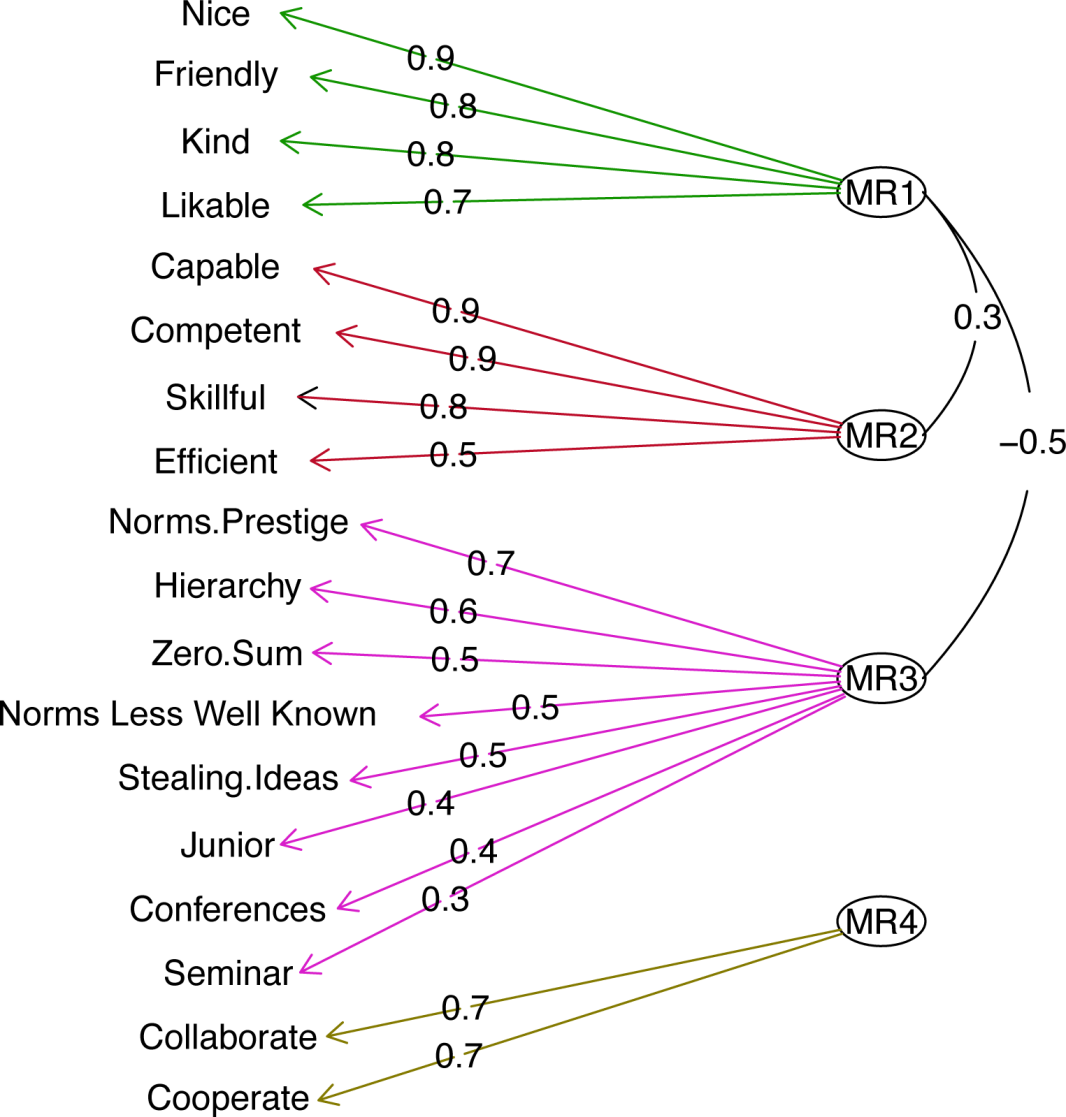
Path diagram showing the four factors that were discovered using exploratory factor analysis. MR1 included items hypothesized to load onto a warmth factor, MR2 included items hypothesized to load onto a competence factor, MR3 we will call zero-sum/hierarchical and MR4 we will call ‘Cooperation’ (though we do not use this as a factor in subsequent analyses).

However, the results did not perfectly discriminate between H1 and H2. H1 predicted a somewhat different structure to the data than the one yielded by the factor analysis. Specifically, H1 predicted that answers to items 7 and 8 (above) would load onto a ‘Competitiveness’ factor with questions 9 and 10. H2 predicted that all items would load onto a single evaluative factor. Partially consistent with both of these hypotheses, the factor analysis grouped items 7 and 8 with items 1−6, which we had hypothesized reflects ‘Hierarchy’. Reliability for the zero-sum/hierarchical factor including these eight items was acceptable (McDonald’s ω = 0.77, 95% CI: 0.72–0.8; λ2 = 0.77, 95% CI: 0.72–0.81). Contrary to H1, McDonald’s ω for the second factor, which included the two questions about cooperation and collaboration, was poor (McDonald’s ω = 0.66, 95% CI: 0.54–0.75; λ2 = 0 .66, 95% CI: 0.54−0.75). Contrary to H2, reliability for all social dynamics questions was lower than the factor with only eight items (McDonald’s ω = 0.74, 95% CI 0.69–0.78; λ2 = 0 .75, 95% CI: 0.71−0.79). In all analyses below, we combine participants' answers to questions 1−8 into one factor that captures perceptions of hierarchy and zero-sum interactions (refer to figure 2).

With regard to judgments of the traits of individuals in the field, reliability for warmth and competence factors were excellent (Warmth: McDonald’s ω = 0.9, 95% CI: 0.88−0.92; λ2 = 0.90, 95% CI: 0.88−0.92; Competence: McDonald’s ω = 0.86, 95% CI: 0.83–0.89; λ2 = 0.86, 95% CI: 0.83−0.89). Combining all the items from the warmth/competence scale as well as the social dynamics questions into a single factor led to a very poor fit (McDonald’s ω = 0.13, 95% CI: 0.015–0.30; λ2 = 0.61, 95% CI: 0.54–0.66). Thus, McDonald’s ω and λ2 for the subscales were higher than a factor that included all the items. Refer to electronic supplementary material for histograms showing how participants answered each question, as well as exploratory analyses of predictors of participants’ perceptions of their field in terms of zero-sum/hierarchical and cooperation/collaboration dimensions (electronic supplementary material, sections 1.1.2 and 1.2).

### 2.3. Study 1 discussion

In Study 1, we found evidence rejecting the null hypothesis H3 and partially supporting H1: participants’ answers to the questions about the social dynamics of their field loaded onto two factors, though only one of them showed good/acceptable reliability. Researchers appear to coherently perceive the social dynamics of their field in terms of the importance of hierarchy and zero-sum competition on the one hand and the importance of collaboration and cooperation on the other. Answers to these questions were distinct from the perceived warmth and competence of individuals in the fields. Using the social dynamics scales developed in Study 1, we next test our main hypothesis that researchers’ perceptions of social dynamics in their field will predict their attitudes to open science.

## 3. Study 2

For Study 2 (pre-registered https://osf.io/h9up8), we emailed researchers in the school of science at a large research-focused university inviting them to answer a survey (refer to figure 1). To test whether researchers' perceptions of social dynamics predict open science attitudes, we performed a mixture of pre-registered confirmatory and exploratory analyses.

### 3.1. Study 2 participants

Participants were recruited via an email that was sent out to graduate students, postdocs, researchers and P.I.s in the school of science at a large research-focused university (exactly 1000 people). The recruitment email described the study as an ‘anonymous survey about the social dynamics in your discipline and open science’ that ‘will be used to help guide policies and practices around open science’. Although 579 researchers initiated the survey, only 271 completed the survey. We collected the same demographic information as in Study 1 (refer to figure 1). Whereas most of the participants in Study 1 were from Psychology, Communications and related fields, the participants in Study 2 were from Chemistry, Biology and Physics. The stopping rule we pre-registered and followed was that we would stop data collection after we sent out three reminders on 9 June 2022, 16 June 2022 and 23 June 2022.

### 3.2. Study 2 materials

We used the same social dynamics survey questions we used in Study 1 and added questions about participants’ attitudes about open science, their open science practices, their underlying motivations to engage in open science and their ideas about other participants’ motivations to engage in open science (refer to https://osf.io/4g2tu/ for the full survey). This survey did not include items about perceptions of warmth or competence. Below are the items included in our survey. We adapted some of these questions from previous surveys (e.g. [9]). We also piloted this survey in our laboratory (approx. 20 people) and received spoken feedback. From this feedback, we modified some of the surveys to reduce redundancy. For the Open Science Plans questions, we asked researchers to choose the extent to which they agreed with the following statements. For the Perceptions of Open Science questions, participants were asked to choose the extent to which the five following motivations affected behaviour for each of the items below.

#### 3.2.1. Open science plans

(1) Attitudes toward open science
  (a) *It is important that researchers in [participant field] publicly share MATERIALS NEEDED TO REPRODUCE RESULTS (e.g. data, code used to analyse data, stimuli).*
  (b) *It is important that researchers in [participant field] publicly share MANUSCRIPTS/PREPRINTS.*
(2) Plans to implement open science
  (a) *For the next piece of research that I publish, I plan to publicly share the MANUSCRIPTS/PREPRINTS.*
  (b) *For the next piece of research that I publish, I plan to publicly share the DATA.*
  (c) *For the next piece of research that I publish, I plan to publicly share the CODE(S) USED TO ANALYSE DATA.*
  (d) *For the next piece of research that I publish, I plan to publicly share the SURVEY INSTRUMENTS OR STIMULI.*

#### 3.2.2. Motivations for open science

(1) *When other researchers in [participant field] publicly share their MANUSCRIPTS/PREPRINTS, how much do you think the following factors play a role in their decision?*
(2) *When other researchers in [participant field] publicly share MATERIALS NEEDED TO REPRODUCE RESULTS in published studies (e.g. data, code used to analyse data and/or stimuli), how much do you think the following factors play a role in their decision?*
(3) *If you plan to publicly share your next MANUSCRIPT/PREPRINT, to what extent do the following motivate you to do so?*
(4) *If you plan to publicly share the MATERIALS NEEDED TO REPRODUCE RESULTS (e.g. data, code used to analyse data and/or stimuli) for your next publication, to what extent do the following motivate you to do so?*
  (a) *Requirements or encouragement from funders or their institution (e.g. the department or university where they work).*
  (b) *Personal benefits such as prestige or citations.*
  (c) *Benefits for [participant field], such as for other researchers in [participant field] who may be able to use the materials for their own work, or to encourage scientific progress in the field.*
  (d) *Benefits for the public, such as increase in public trust of science, or downstream benefits of having reliable or reproducible science.*
  (e) *Requirement or encouragement from the P.I. of laboratory (select N/A if you are the PI of the laboratory) motivated themselves and others to share manuscripts and materials.*
  (f) *N.A.*

### 3.3. Study 2 hypotheses and pre-registered analysis plan

In our pre-registration, we planned to first replicate the factor analyses for the social dynamics questions. Then, we planned to perform exploratory analyses to investigate the structure of the remaining survey items. Finally, we planned to perform exploratory analyses about whether participants’ attitudes about the social dynamics in their field and their perceptions of open science predicted their open science plans.

### 3.4. Study 2 results

#### 3.4.1. Social dynamics

In pre-registered analyses, we replicated the findings from Study 1. Confirmatory factor analysis organized the items along the same factors as in Study 1 (χ^2^ = 122.51, *p* < 0.001); reliability was ‘good/acceptable’ for the zero-sum/hierarchical factor (McDonald’s ω = 0.76, 95% CI: 0.73−0.80; λ2 = 0.76, 95% CI: 0.73−0.80). Reliability for the cooperation/collaboration items was again poor (McDonald’s ω = 0.56, 95% CI: 0.36−0.59; λ2 = 0.49, 95% CI: 0.36−0.59). Thus, we replicated the finding that questions 1−8 about social dynamics load onto one factor and the other two did not. When computing Bayes factors comparing BICs, we found that 2 factors had the most evidence (Comparing 2 factors to 1: BF₁₀ = 82; Comparing 2 factors to 3: BF₁₀ = 11). This last analysis was not pre-registered and was performed after a suggestion from a reviewer. Refer to electronic supplementary material, for histograms (electronic supplementary material, section 2.1.1), a correlation matrix (electronic supplementary material, section 2.1.3) and additional analyses of answers to the social dynamics questions (electronic supplementary material, section 2.1.4).

#### 3.4.2. Plans to practice open science and ideas about its importance

Next, in pre-registered analyses, we used exploratory factor analysis to determine the structure of participants’ attitudes to open science (refer to electronic supplementary materials for histograms of participant’s answers (electronic supplementary material, section 2.3.1)). The exploratory factor analysis yielded 2 factors. Plans to share materials (materials, data, code and instruments) and the importance of sharing such materials loaded onto one factor. Plans to share manuscripts and the importance of sharing manuscripts loaded onto another (χ^2^ = 29.85, *p* < 0.001; BF₁₀ for 2 versus 1 factor is 187). We were unable to compare models with 2 and 3 factors because the 3-factor analysis did not yield a BIC and the Tucker–Lewis Index of factoring reliability was negative infinity.

Reliability analyses, however, called into question how we should separate the factors. McDonald’s ω for the factor about manuscripts was 0.72 (95% CI: 0.63−0.79) and λ2 = 0.72 (95% CI: 0.63−0.79). McDonald’s ω for the factor about scientific materials was 0.74 (95% CI: 0.70−0.80) and λ2 = 0.72 (95% CI: 0.66−0.79). McDonald’s ω for one factor that combines all the items was 0.68 (95% CI: 0.62−0.74) and λ2 = 0.71 (95% CI: 0.65−0.76). Because reliability did not differ substantially (95% CIs overlapped) between these different structures, we combined answers to all questions about open science into a single weighted factor, called ‘Open science attitudes’.

#### 3.4.3. Motivations to practice open science

We also asked participants why they practice open science and why they think others practice open science (refer to electronic supplementary material, section 2.4.1 for histograms of participants’ answers to these questions). We asked participants to rate the importance of each motivation for their own decisions to share manuscripts and materials, as well as for other researchers in their field.

Since we did not have hypotheses ahead of time about the structure of this data, we used exploratory factor analysis to investigate the structure of the data. Using this method, we found evidence for 5 factors (χ^2^ = 973.1, *p* < 0.001). The first factor was requirements/encouragement from one’s laboratory or P.I. There was high reliability between four questions about motivations based on encouragement or requirements from a person’s laboratory or P.I. as reasons for self and others to share materials and manuscripts (McDonald’s ω = 0.85, 95% CI: 0.81−0.89; λ2 = 0 .85, 95% CI: 0.81−0.89). The second factor was the public good. There was high reliability for the four questions about motivations based on the public good as reasons for self and others to share materials and manuscripts (McDonald’s ω = 0.86, 95% CI: 0.83–0.89; λ2 = 0.86, 95% CI: 0.84−0.89). The third factor was requirements and encouragement from funding or journals. There was high reliability between the four questions about motivations about requirements or encouragement from funding or journals as reasons for self and others to share materials and manuscripts (McDonald’s ω = 0.82, 95% CI: 0.79−0.86; λ2 = 0.82, 95% CI: 0.78−0.85). The fourth factor was prestige. There was medium reliability between the four questions about motivations about prestige as a reason for self and others to share materials and manuscripts (McDonald’s ω = 0.80, 95% CI: 0.76–0.83; λ2 = 0.90, 95% CI: 0.75−0.83).

Unlike the first four factors, for cooperation with other researchers, people’s answers loaded onto separate factors for self and others. There was acceptable reliability for the two items measuring self-motivation to cooperate with other researchers by sharing materials and manuscripts (McDonald’s ω = 0.69, 95% CI: 0.60−0.76; λ2 = 0.69, 95% CI: 0.60−0.76). There was acceptable reliability for the two items about others’ motivations to cooperate with other researchers by sharing materials and manuscripts (McDonald’s ω = 0.67, 95% CI: 0.54−0.76; λ2 = 0.67, 95% CI: 0.54−0.76).

We used BIC to compute Bayes factors, which compared a model with these 5 factors, to models with 1, 2, 3, 4, 6 and 7 factors (note that this was not pre-registered and was done after a reviewer suggested it). We found evidence that the data fit a model with 5 factors better than models with 1, 2, 3 or 4 factors, but not 6 or 7 (BF₁₀ for 5 factors compared to 1: BF₁₀ = 5.3 × 10^145^; BF₁₀ for 5 factors compared to 2: BF₁₀ = 6.7 × 10^61^; BF₁₀ for 5 factors compared to 3: BF₁₀ = 2.7 × 10^37^, BF₁₀ for 5 factors compared to 4: BF₁₀ = 4 × 10^^16^; BF₁₀ for 6 compared to 5 factors was 3.52 × 10^9^ BF₁₀ for 7 compared to 5 was 7.18 × 10^15^).

This factor analysis does not directly lend evidence one way or another to our hypotheses. We performed this factor analysis to refer to whether we should combine items. Since the cooperation and prestige motivations were relevant to the hypotheses those questions are used in the analyses at the end of this section. The analysis informed how we used the answers to these questions in the models at the end of this section—combining self and other answers for prestige and keeping them separate for cooperation.

Before we turn to those models and because the cooperation loaded on distinct factors, when answering about one’s own versus other researchers’ motivations for open science, we next asked whether people think that others have different reasons for practising open science than themselves.

#### 3.4.4. Exploratory analysis: do participants think that other people are motivated to share for different reasons than themselves?

We pre-registered that we would ask whether people answered the questions about motivation differently about themselves and other people, but we did not pre-register the details of this analysis. We used the package brms [31] to compare a model that included an interaction between the specific motivation and whether the participants were answering about themselves or others and a model that did not include this interaction.

**Figure 3.**
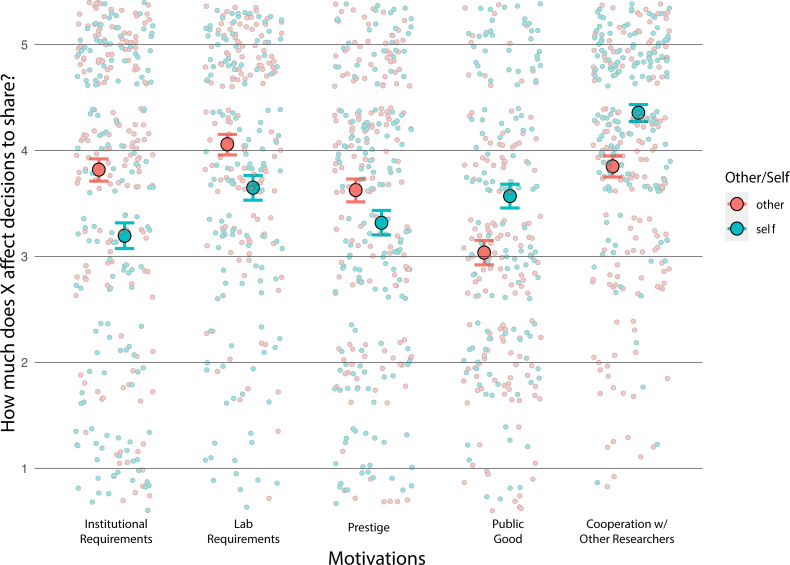
From Study 2. Scatter plot and 95% credible intervals for self versus other for each motivation, ‘*When other researchers/you in [participant field] publicly share their/your [scientific product], how much do you think the following factors play a role in their/your decision?’* Large circles are predicted means, and small faint circles are individual data points. The *x*-axis is the different motivational factors we asked about. The *y*-axis reflects the Likert scale used in the survey that ranged from ‘not at all’ to ‘a great deal’. Here, we found that participants said other researchers are more motivated by institutional requirements, laboratory requirements and prestige, while they reported that their own motivations were the public good and to cooperate with other researchers.

When participants reasoned about themselves compared to others, they answered the questions differently (BF₁₀ = 1.22 × 10^65^ in favour of a model that includes the interaction; refer to figure 3). Participants said other researchers are more motivated by institutional requirements, laboratory requirements and prestige. Participants said they are more motivated by the public good and to cooperate with other researchers than other researchers.

#### 3.4.5. Exploratory analysis: do participants’ ideas about social dynamics predict open science attitudes?

Our goal was to test whether participants’ perceptions of the social dynamics in their field, their perceptions of motivations to practice open science and/or an interaction between the two predicted their open science attitudes. We hypothesized that people who viewed their field as more zero-sum/hierarchical would have less favourable attitudes to open science. We also hypothesized that people who said they and others shared to cooperate would have a more favourable view of open science, and those who shared because of prestige would have a less favourable view of open science. Finally, we predicted that there could be an interaction between these two factors. Specifically, the less researchers saw their field as zero-sum/hierarchical, and the more open science was motivated to cooperate with others, the more favourable their attitudes to open science would be. Conversely, the more researchers saw their field as zero-sum/hierarchical, and the more open science was motivated by prestige, the more positive their attitudes to open science would be. The specific analyses used here are exploratory because we determined the specific analytic strategy after the data were collected. All analyses were repeated in an independent sample in Study 3.

In each case, we used the package brms to fit models. All model fits were checked using the pp_check (posterior predictive checks) function in brms (this allows a researcher to check whether the model is reasonably capturing the data through visual inspection; refer to electronic supplementary materials for the results of these checks, electronic supplementary material, section 2.6.1.1). We plot estimated means and 89% credible intervals and consider any factor whose 89% CI does not include zero to be meaningful (these are analogous to confidence intervals in frequentist analyses; [32]). We chose 89% because it is standard in Bayesian analyses. We report these means and credible intervals. We had originally planned to compute Bayes factors but decided against it because we were getting divergent models for many of our null models, so the Bayes factors would not be meaningful. Since our hypotheses were primarily based on people’s perceptions of open science as either a means of cooperation or gaining prestige and our sample size was small, we only included these two motivations in our models. All tests were non-directional.

First, we tested a model that asked whether people’s perceptions of social dynamics in their field predicted their open science attitudes. The outcome variable was a weighted average of participants’ answers to the open science questions. As predictors, the model included participants’ scores on the zero-sum/hierarchical questions (a weighted sum according to the factor analysis performed above), participants’ answers to each of the collaboration and cooperation questions separately, how much participants identified with their field and the participants’ years of experience in the field (log-transformed).

**Figure 4.**
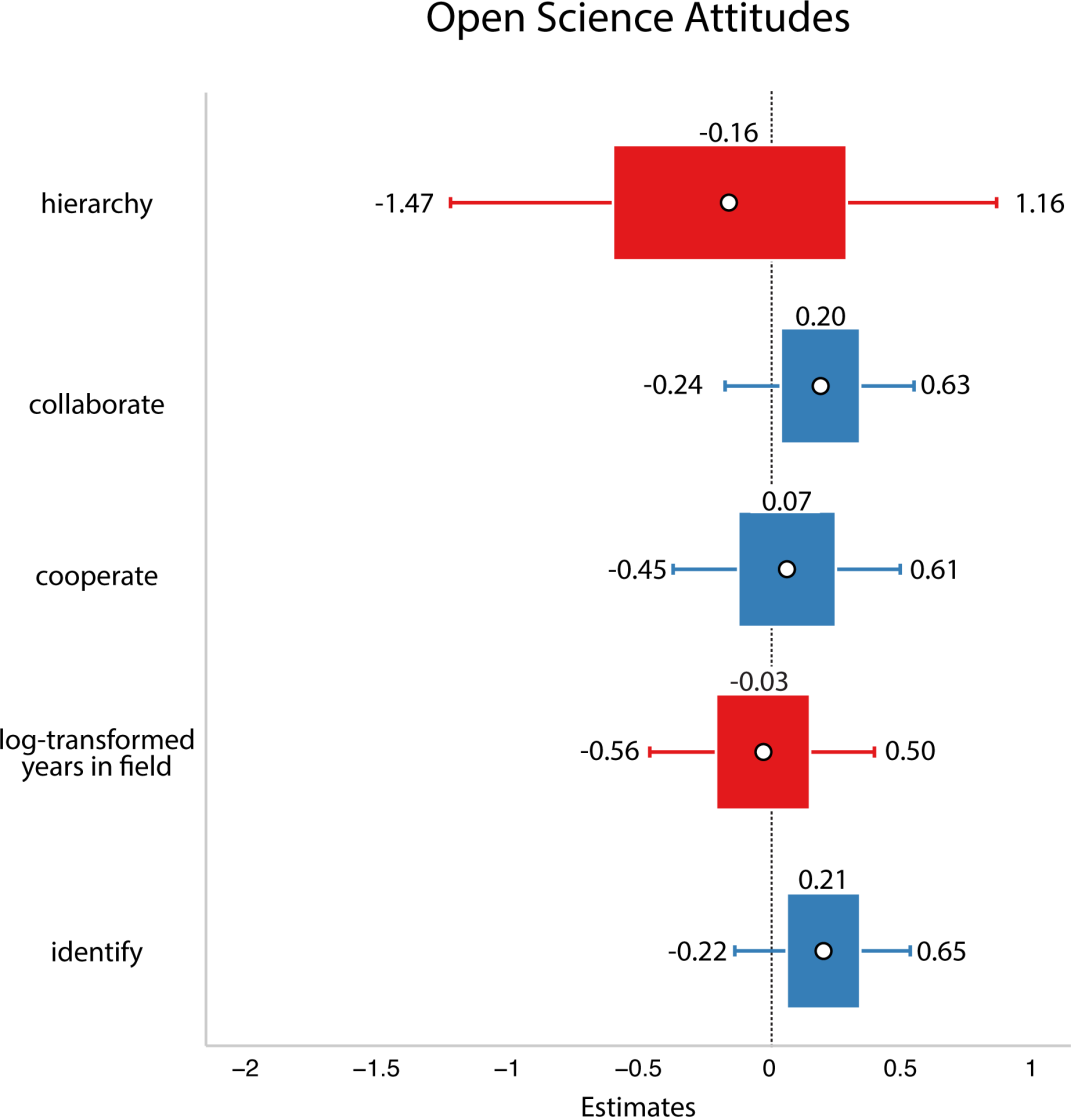
Study 2 data. White circles are estimated means, inner bars are 50% highest density intervals, outer bars are 89% highest density intervals plotted using the R package sjPlot [33]. The outcome variable was open science attitudes. Here, we did not find evidence that any of the social dynamics affected people’s open science attitudes.

In this analysis, we did not find support for our hypothesis: we did not find evidence that researchers who viewed their field as more zero-sum/hierarchical have less favourable attitudes to open science (refer to figure 4).

#### 3.4.6. Exploratory analysis: do participants’ motivations and perceptions of other researchers’ motivations predict open science attitudes?

Next, we asked whether people’s answers to the motivation questions predicted their open science attitudes. As predictors, we used the weighted sum of the prestige questions (for both self and other) and the cooperation questions (separately for self and other), based on the factor analysis above. We did not include laboratory encouragement/requirements (both self and other), institution encouragement/requirements (both self/other) or public good (both self and other) questions.

**Figure 5.**
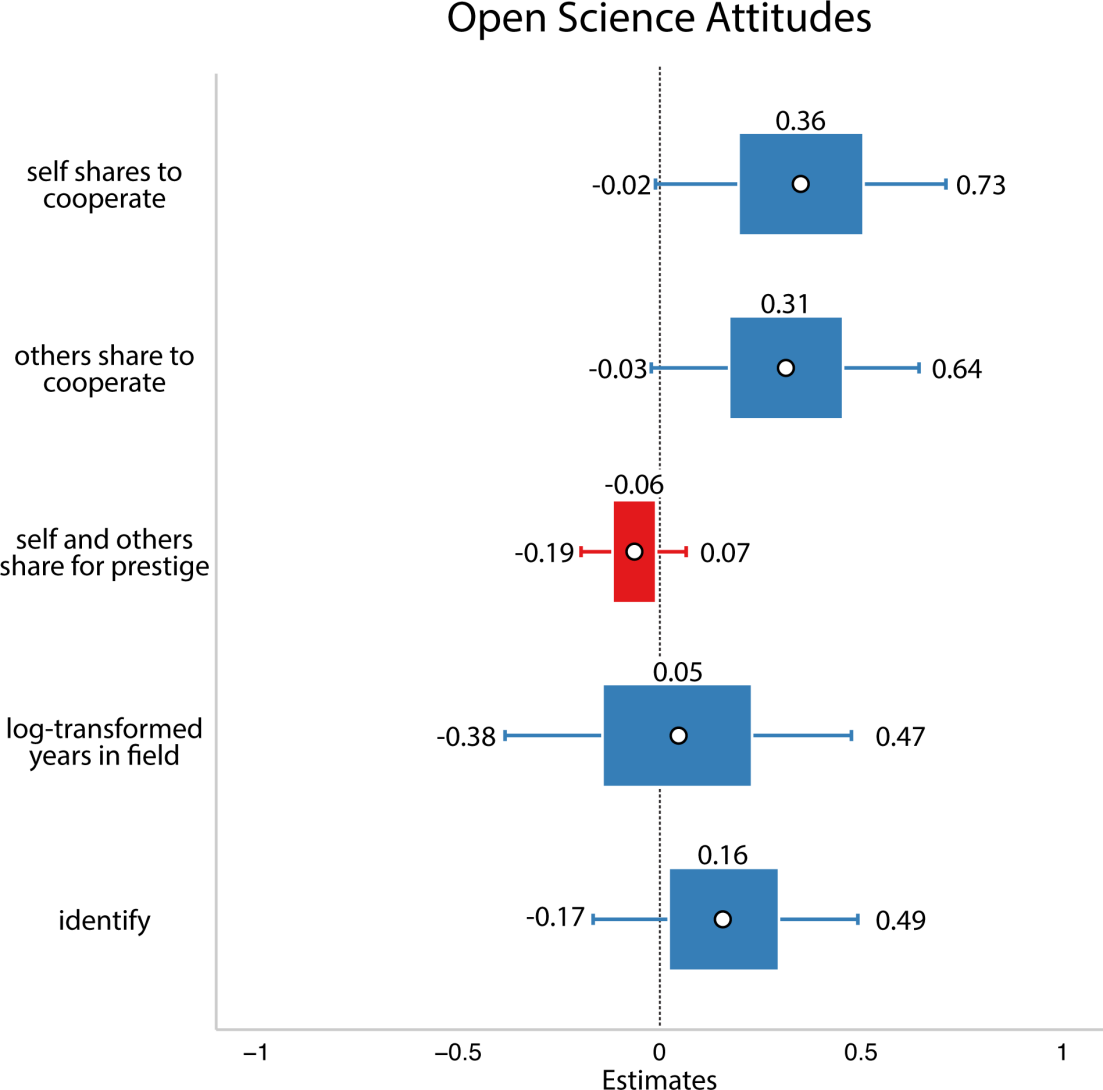
Results from Study 2. White circles are estimated means, inner bars are 50% highest density intervals and outer bars are 89% highest density intervals. Plotted using the R package sjPlot [33]. The outcome variable was open science attitudes. Here, we did not find that reported or perceived motivations predicted people’s attitudes about open science.

In this analysis, there were trends in the hypothesized direction: researchers who said they and others share to cooperate with other researchers seemed to be trending towards having more positive open science attitudes. However, we interpret these cautiously since the 89% credible intervals include zero (refer to figure 5).

#### 3.4.7. Exploratory analysis: does the fit between perceived motivations and perceived social dynamics predict open science attitudes?

We predicted that the more people saw open science as cooperative and the more they saw their field as cooperative, the more likely they would be to engage in open science. Therefore, we tested a model that included interactions between the zero-sum/hierarchical factor and whether researchers said they, or others, engage in open science practices to cooperate with other researchers. Our hypothesis predicted a negative interaction: the less researchers saw their field as zero-sum/hierarchical and the more open science was motivated to cooperate with others, the more favourable their attitudes to open science would be.

**Figure 6.**
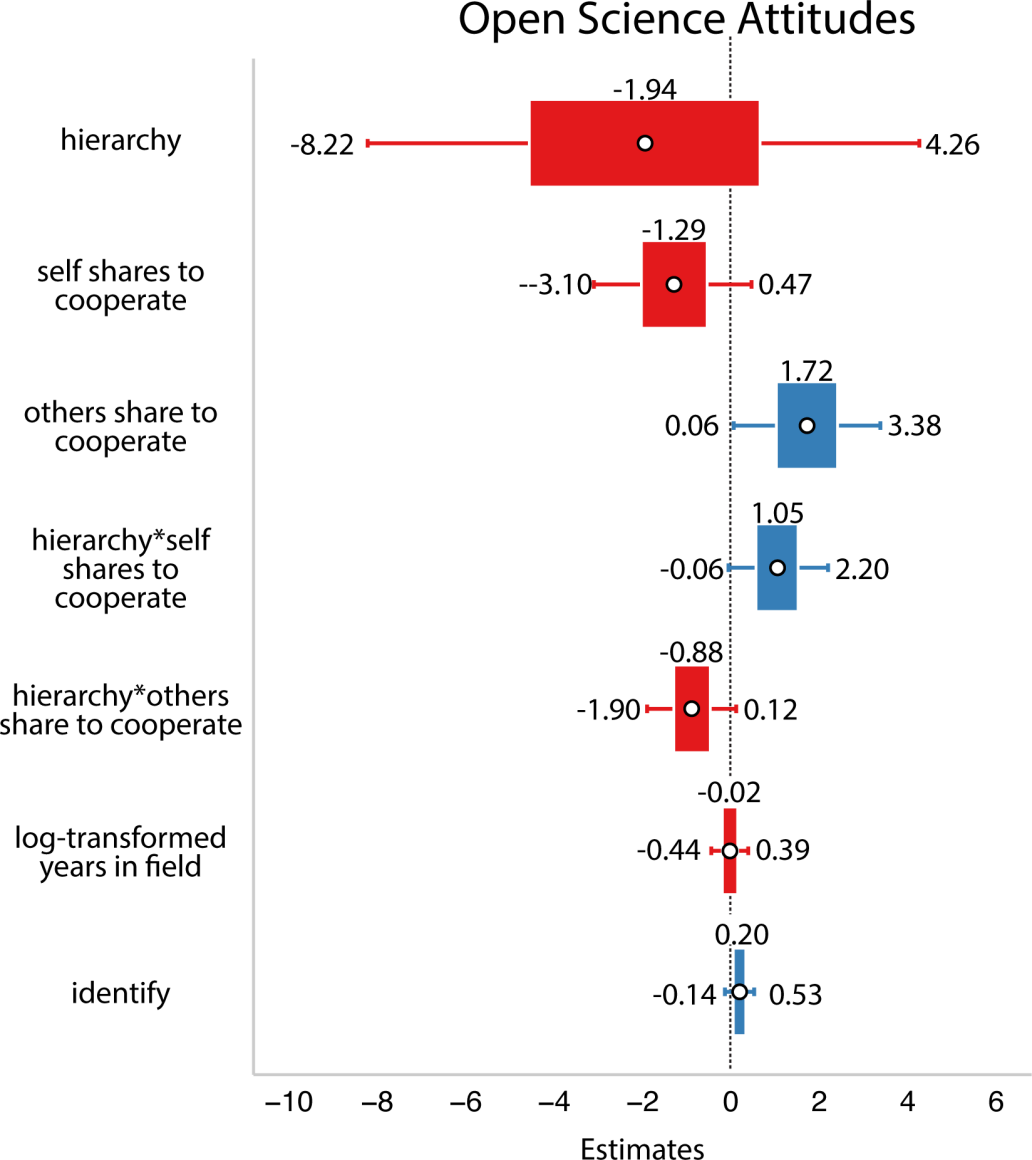
Results from Study 2. White circles are estimated means, inner bars are 50% highest density intervals and outer bars are 89% highest density intervals. Plotted using the R package sjPlot [33]. The outcome variable was open science attitudes. After accounting for potential interactions with social dynamics, participants who said that *others*’ motivation for open science was to cooperate expressed stronger positive attitudes towards open science practices.

In this analysis, after accounting for potential interactions with social dynamics, researchers who said that *others*’ motivation for open science was to cooperate expressed stronger positive attitudes towards open science practices themselves. In addition, there was a trend toward the predicted negative interaction: the influence of others’ perceived motivations on open science attitudes tended to be stronger among researchers who saw their field as *less* zero-sum/hierarchical (refer to figure 6). On the other hand, we observed the opposite trends for researchers who reported their own motivation for open science was to cooperate. For the items measuring one’s own motivation to cooperate, there was no main effect or interaction with social dynamics and the trends appeared in the opposite direction of the effects of others’ motivations to cooperate.

**Figure 7.**
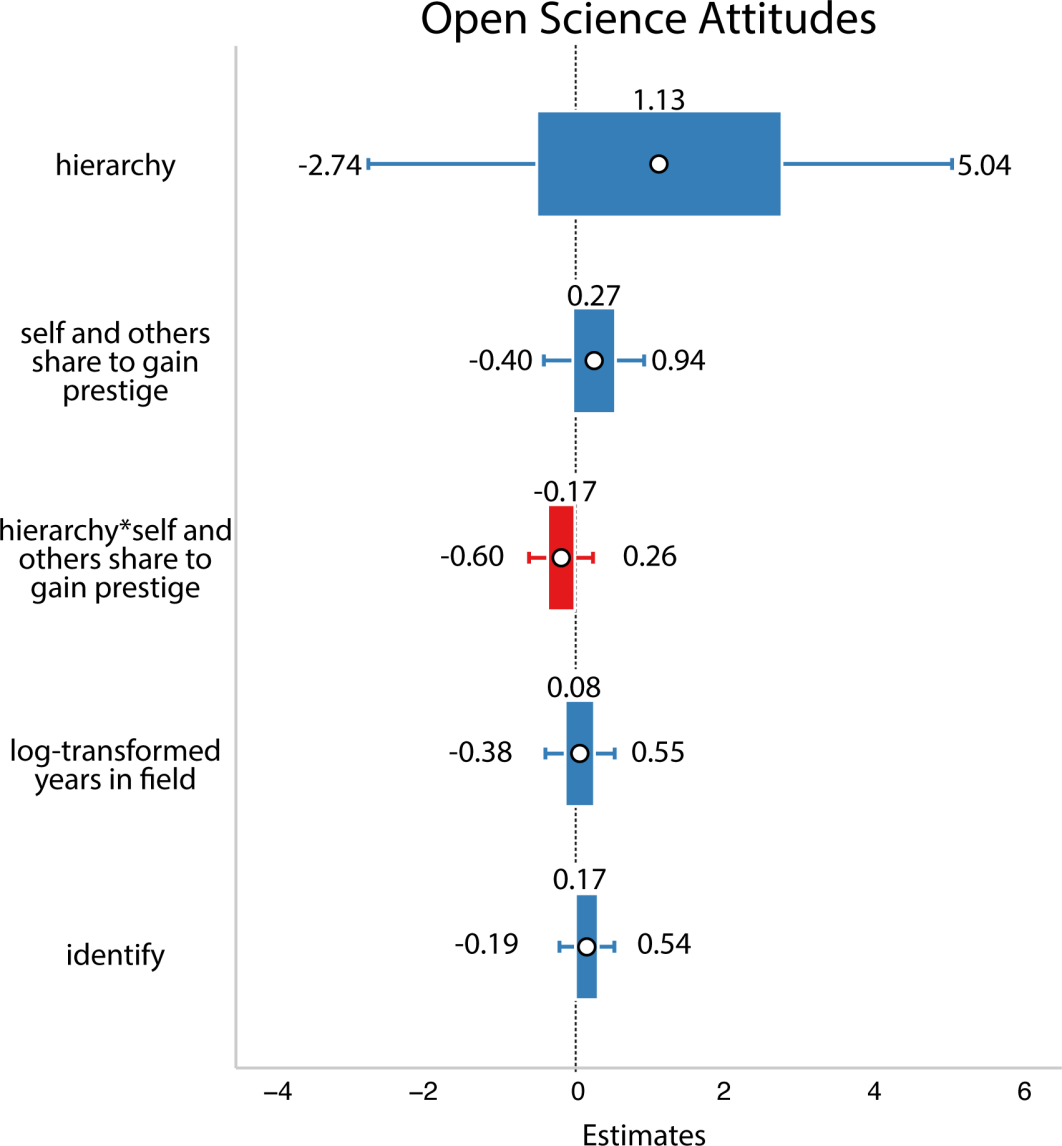
Results from Study 2. White circles are estimated means, inner bars are 50% highest density intervals and outer bars are 89% highest density intervals. Plotted using the R package sjPlot [33]. The outcome variable was open science attitudes Here, we found no evidence that people’s perception of social dynamics and motivations to gain prestige interacted.

Conversely, we predicted that the more people saw open science as a way to gain prestige and the more that they saw their field as competitive, the more likely they might be to engage in open science. Therefore, we tested a model that included interactions between the zero-sum/hierarchical factor and whether researchers said they, or others, engage in open science practices for prestige. Our hypothesis predicted a positive interaction between zero-sum/hierarchical social dynamics and motivations for prestige. However, we found no evidence for this hypothesis (figure 7).

### 3.5. Study 2 discussion

In Study 2, we replicated the finding from Study 1 that researchers’ answers to questions about social dynamics relate to one another, suggesting they have a coherent view of social dynamics. Furthermore, people’s self-reported motivations for sharing manuscripts and materials differ from their perceptions of why others share manuscripts and materials. Researchers see themselves as more motivated by prosocial motives (cooperating with other researchers, public good) and others as more motivated by requirements or prestige. This pattern is consistent with longstanding evidence of self-serving biases in explanations of one’s own versus others’ behaviour [34–36].

Next, in exploratory analyses, we tested our key novel question: whether perceptions of social dynamics, people’s reasons for sharing and perceptions of other people’s reasons for sharing predicted participants’ plans for and attitudes about sharing manuscripts and materials. We did find some trends that were in line with our hypotheses. In particular, people who said others shared to cooperate were more likely to have positive open science attitudes. However, we did not observe the predicted effects of perceived social dynamics.

One reason to be cautious in interpreting these null results is that the participants in Study 2 were self-selecting and our analyses were exploratory. Among participants who volunteered to take a survey about open science, we found high rates of support for open science. For example, on the questions that we asked about support for open science, participants' average score was over 4 (on a scale from 1 to 5) and the median answer was 5 (refer to electronic supplementary materials for more details). The limited variance in support for open science may reduce the power of this study to detect predictors of such support. Therefore, in the final confirmatory study, we repeat the same analyses with a more representative sample of science researchers. This final confirmatory study was submitted as a registered report (Stage 1 accepted manuscript can be found here: https://osf.io/w5g92).

## 5. Study 3

In Study 3, we tested the same hypotheses, in a representative sample of researchers with a very high rate of participation (refer to [37] for more details on how people’s support for open science compared across these studies). As a reminder, we predicted that people’s attitudes about the social dynamics of their field and their perceptions of open science would predict their open science plans. For example, the more that people thought that their field was hierarchical/zero-sum and the more they thought that open science practices were a way to cooperate with others, the less they would support open science. This sample drew from a private research-focused institution that is not necessarily representative of other universities in the region. The only relevant institute-wide policy at the time of data collection was a Faculty Open Access policy that grants the institution the right to openly disseminate faculty journal articles. However, faculty could ‘opt out’ of the policy on an article-by-article basis. Like many places, there were active discussions about the institute’s role in promoting open science at the time of data collection. Prior to data collection, we expected that we would find more variation in people’s attitudes to open science than in Study 2, because of the difference in sampling approaches. We performed the same analyses that we performed in Study 2 on this new sample.

### 5.1. Study 3 participants

To recruit participants, we contacted 35 principal investigators in the School of Science at the same university as in Study 2, by asking if we could join a laboratory meeting to discuss priorities for the Dean’s office around supporting science in general and open science in particular. We scheduled visits to 26 laboratory meetings, approximately 5 per department, over Zoom during 2021. The data were collected before we submitted the Registered Report, but the researchers did not access the data prior to receiving an in-principle acceptance. The total number of people attending these laboratory meetings was 315, and 299 (94.9%) people completed the survey. We also asked about how many people were missing from the laboratory meeting (that is people who regularly attended who were not there, a total of 22). Most participants did not know the purpose of the laboratory meeting ahead of time. Thus, the sample of respondents was representative of laboratory meeting attendees, regardless of interest in or attitudes towards open science.

### 5.2. Study 3 materials

We used the same survey as described in Study 2.

### 5.3. Study 3 results

For all analyses, we included the same items into scales for perceived social dynamics, perceived motivations for open science and attitudes to open science, derived from Study 2. For our confirmatory analyses, we used the exact weights derived from Study 2. In confirmatory analyses, we investigated how similar the structure of correlations within these items was in Study 3 compared to Study 2. In exploratory analyses, we used weights derived from the factor analysis performed in Study 3 to investigate whether the outcomes differ across all models.

#### 5.3.1. Confirmatory analysis: do the factors discovered in Study 2 fit the structure of variance in responses in Study 3?

We ran a confirmatory factor analysis and compared the weights from Study 2 and Study 3 for the items about social dynamics. We found that the factor analysis yielded two factors with all items loading onto one factor except for the questions about collaboration and cooperation (evidence for 2 factors over 1, BF₁₀ = 5165; evidence in favour of 2 over 1 factor; BF₁₀ = 221 727). Therefore, we found the same structure in Study 3 as in Study 2.

#### 5.3.2. Confirmatory analysis: do researchers think that other people are motivated to share for different reasons than themselves?

As in Study 2, to investigate whether people give different explanations as to why they practice open science compared to others, we used the package brms [31] to compare a model that included an interaction between the specific motivation and whether the participants were answering about themselves or others and a model that did not include this interaction.

**Figure 8.**
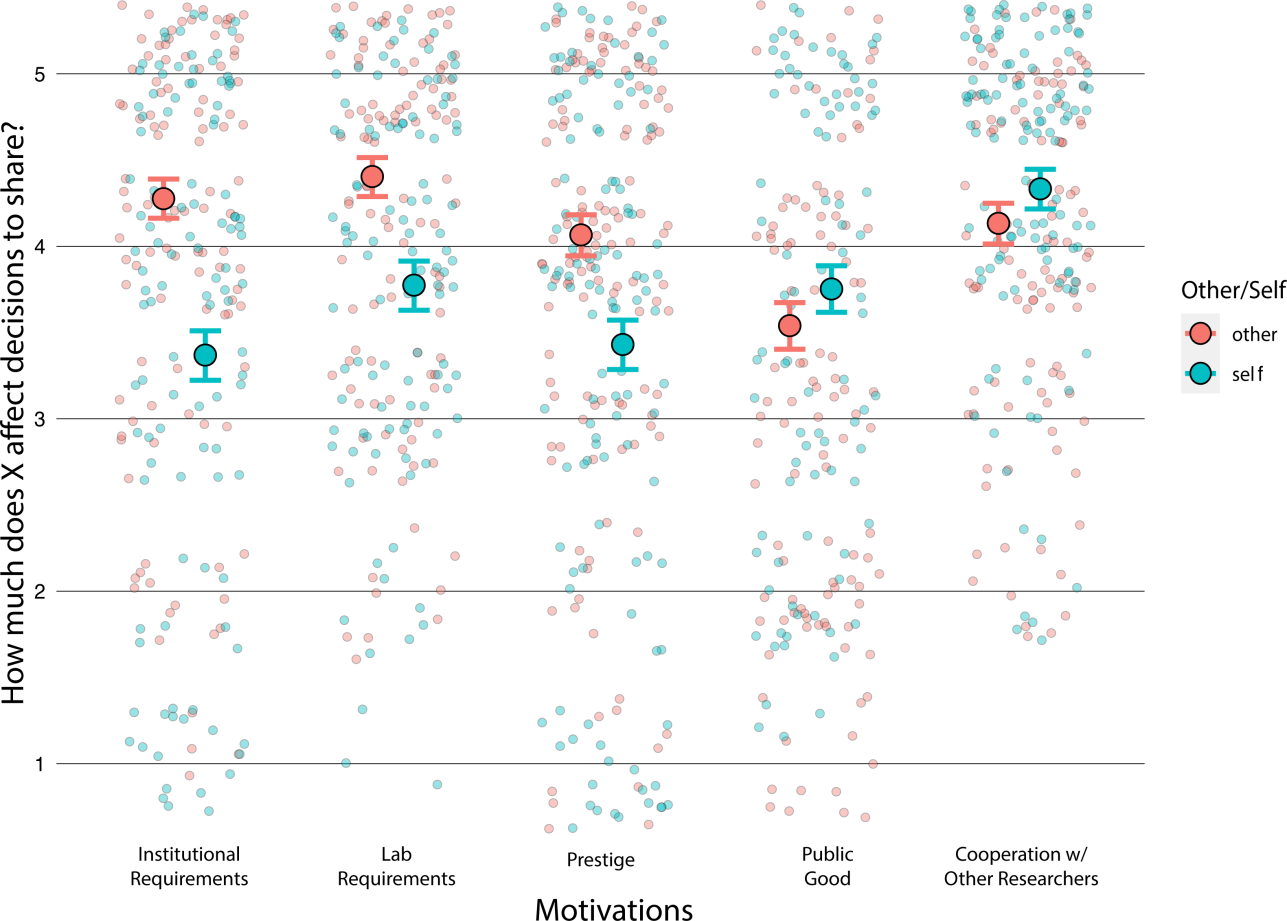
Results from Study 3. Scatter plot and 95% credible intervals for ratings of self and other motivations, ‘*When other researchers/you in [participant field] publicly share their/your [science product], how much do you think the following factors play a role in their/your decision?’* Large circles are predicted means and small faint circles are individual data points. The *x*-axis is the different factors we asked about. The *y*-axis reflects the Likert scale used in the survey from ‘not at all’ to ‘a great deal’. Here, we replicated the result from Study 2 and found that participants said other researchers are more motivated by institutional requirements, laboratory requirements and prestige, while they reported being more motivated by the public good and to cooperate with other researchers.

When participants reasoned about themselves compared to others, they answered the questions differently (BF₁₀ = 3.95 × 10^12^) refer to figure 8. These results agreed with the results of Study 2 confirming that people likely overestimate the degree to which others share because of laboratory requirements, institutional requirements and prestige, since participants say they are motivated more by benefits for the public and to cooperate with other researchers.

Next, we did a series of confirmatory analyses to ask whether participants’ perceptions of the social dynamics in their field, their perceptions of motivations to practice open science and/or an interaction between the two predicted their open science attitudes. In each case, we used the package brms to fit a Gaussian model. All model fits were checked using the pp_check (posterior predictive checks) function in brms (this allows a researcher to check whether the model is reasonably capturing the data through visual inspection, refer to electronic supplementary materials for the results of these checks). We report estimated means and 89% highest density intervals and consider any factor whose high density interval (HDI) does not include zero to be meaningful.

#### 5.3.3. Confirmatory analysis: do researchers’ ideas about social dynamics predict open science attitudes?

As in Study 2, we tested a model that asked whether people’s perceptions of social dynamics in their field predicted their open science attitudes. The outcome variable was a weighted average of participants’ answers to the open science questions. As predictors, the model included participants’ scores on the zero-sum/hierarchical factor computed as a weighted sum, participants’ answers to each of the collaboration and cooperation questions, how much they identified with their field and how many years they had been a researcher (log-transformed).

**Figure 9.**
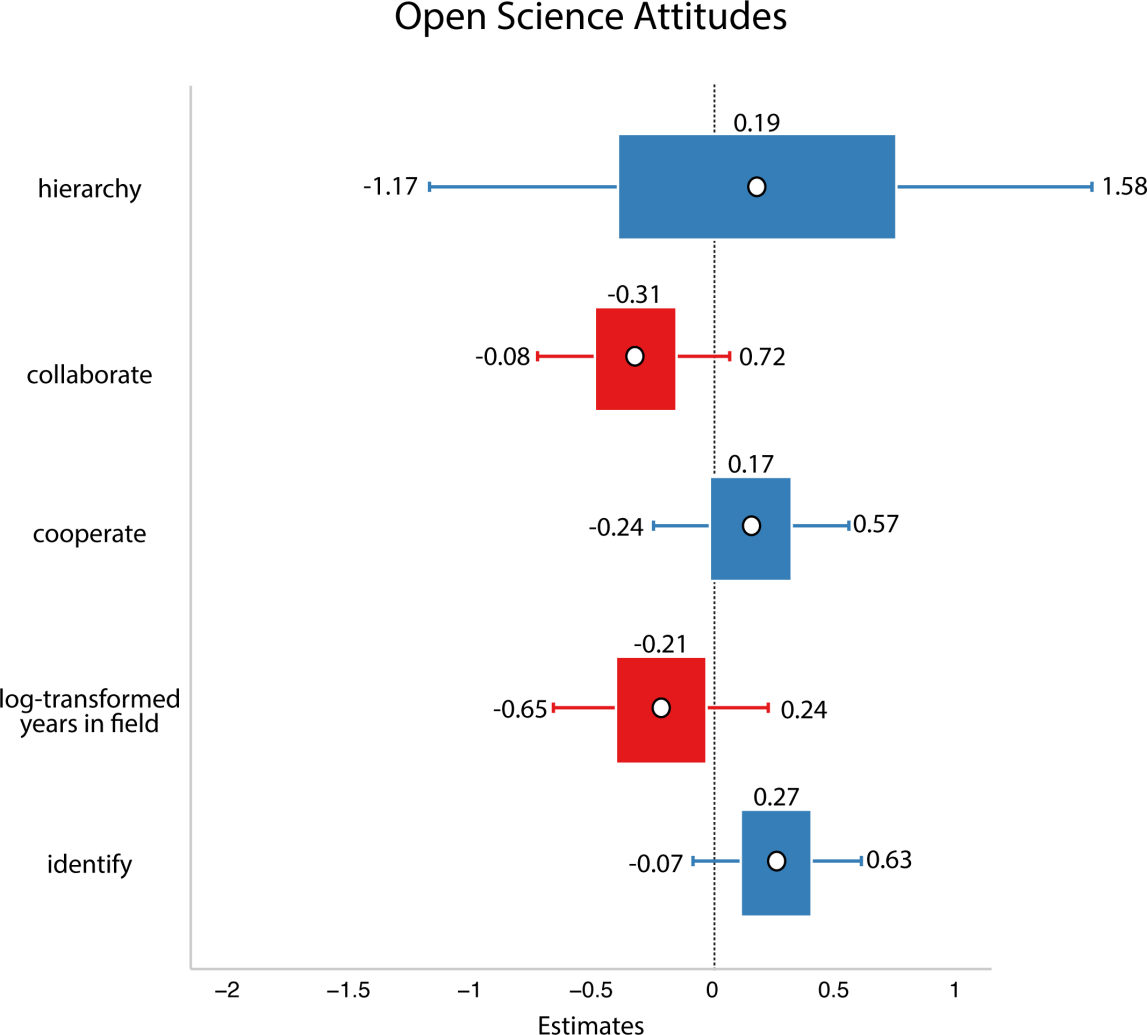
Results from Study 3. White circles are estimated means, inner bars are 50% highest density intervals and outer bars are 89% highest density intervals. Plotted using the R package sjPlot [33]. The outcome variable was open science attitudes. We again found no evidence that perceptions of social dynamics affect people’s open science practices.

In this analysis, we did not find support for our hypothesis: researchers’ ideas about social dynamics did not predict their open science attitudes (refer to figure 9).

#### 5.3.4. Confirmatory analysis: do researchers’ motivations and perceptions of others’ motivations predict open science attitudes?

Next, we asked whether people’s answers to the motivation questions predicted their open science attitudes.

**Figure 10.**
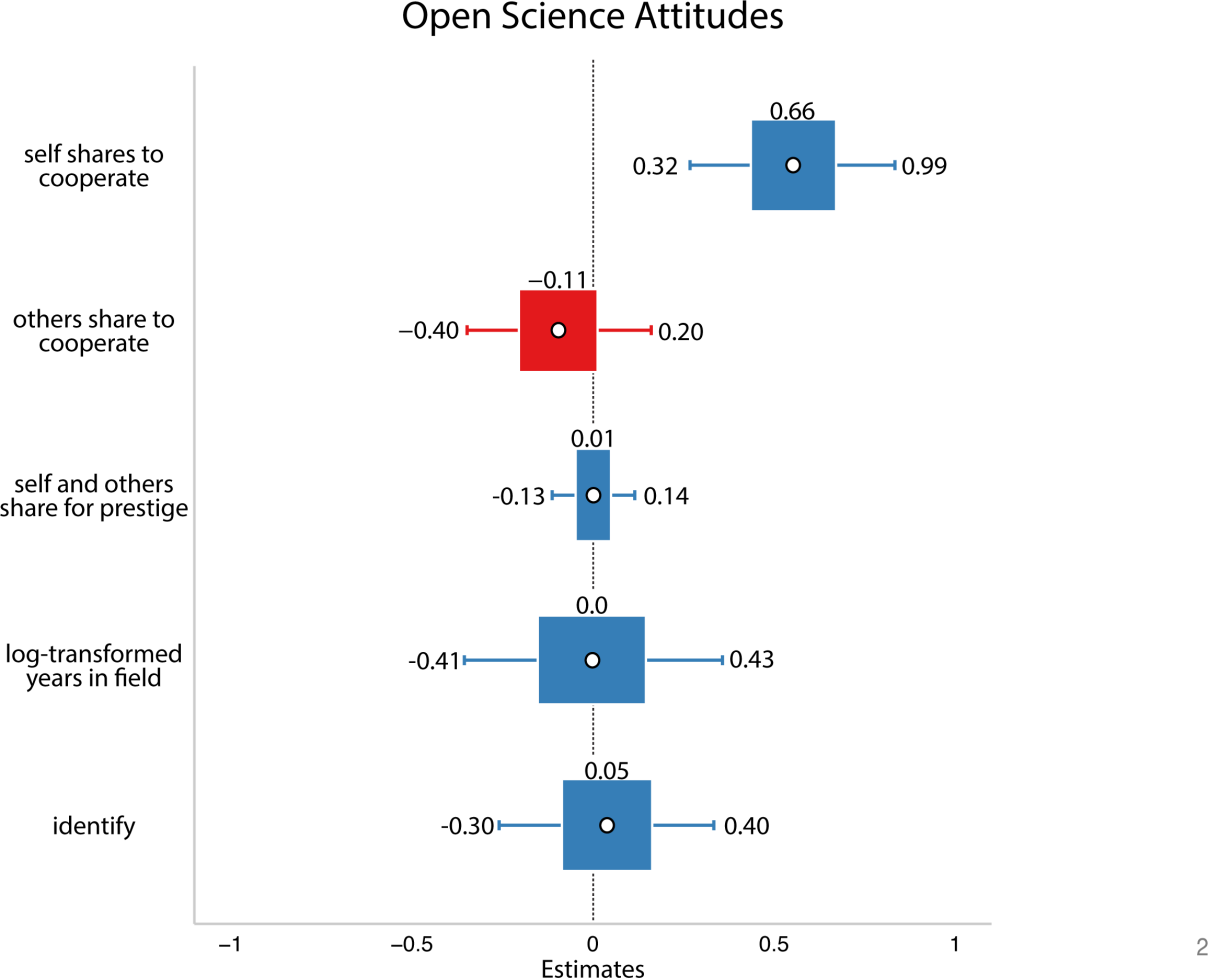
Results from Study 3. White circles are estimated means, inner bars are 50% highest density intervals and outer bars are 89% highest density intervals. The outcome variable was open science attitudes. Here, we found evidence that when people said they share to cooperate they were more likely to endorse open science.

In this analysis, we found some support for our hypothesis that people’s self-reported motivations and perception of other people’s motivations for open science predict their open science attitudes. Here, the more people said they shared to cooperate with others, the more likely they were to say they engaged in open science practices. However, none of the other factors predicted people’s responses (refer to figure 10).

#### 5.3.5. Confirmatory analysis: does the fit between perceived motivations and perceived social dynamics predict open science attitudes?

Finally, we asked whether there was an interaction between participants’ perceptions of open science and the factors about motivations that did predict people’s plans. We predicted that the more people saw open science as cooperative and the less that they saw their field as zero-sum/hierarchical, the more positive their attitudes to open science.

Here, we tested a model that included interactions between the zero-sum/hierarchical factor and whether they, or others, shared to cooperate with other researchers and how much they identify with their field, how many years they have been in their field (log-transformed).

**Figure 11.**
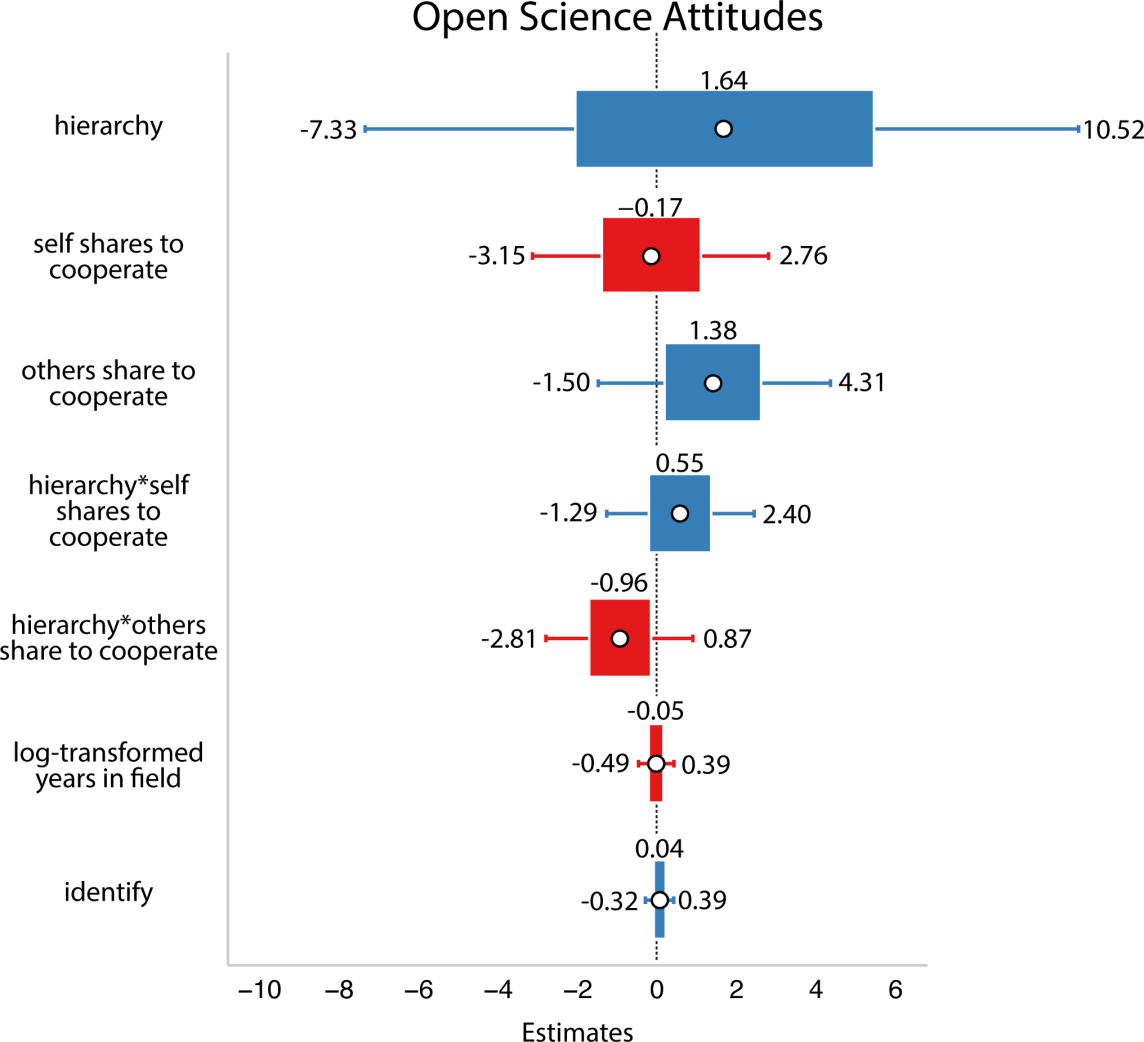
Results from Study 3. White circles are estimated means, inner bars are 50% highest density intervals and outer bars are 89% highest density intervals. Plotted using the R package sjPlot [33]. The outcome variable was open science attitudes. Here, we found no evidence that people’s perceptions of the social dynamics in their field interacted with motivations to predict open science attitudes.

**Figure 12.**
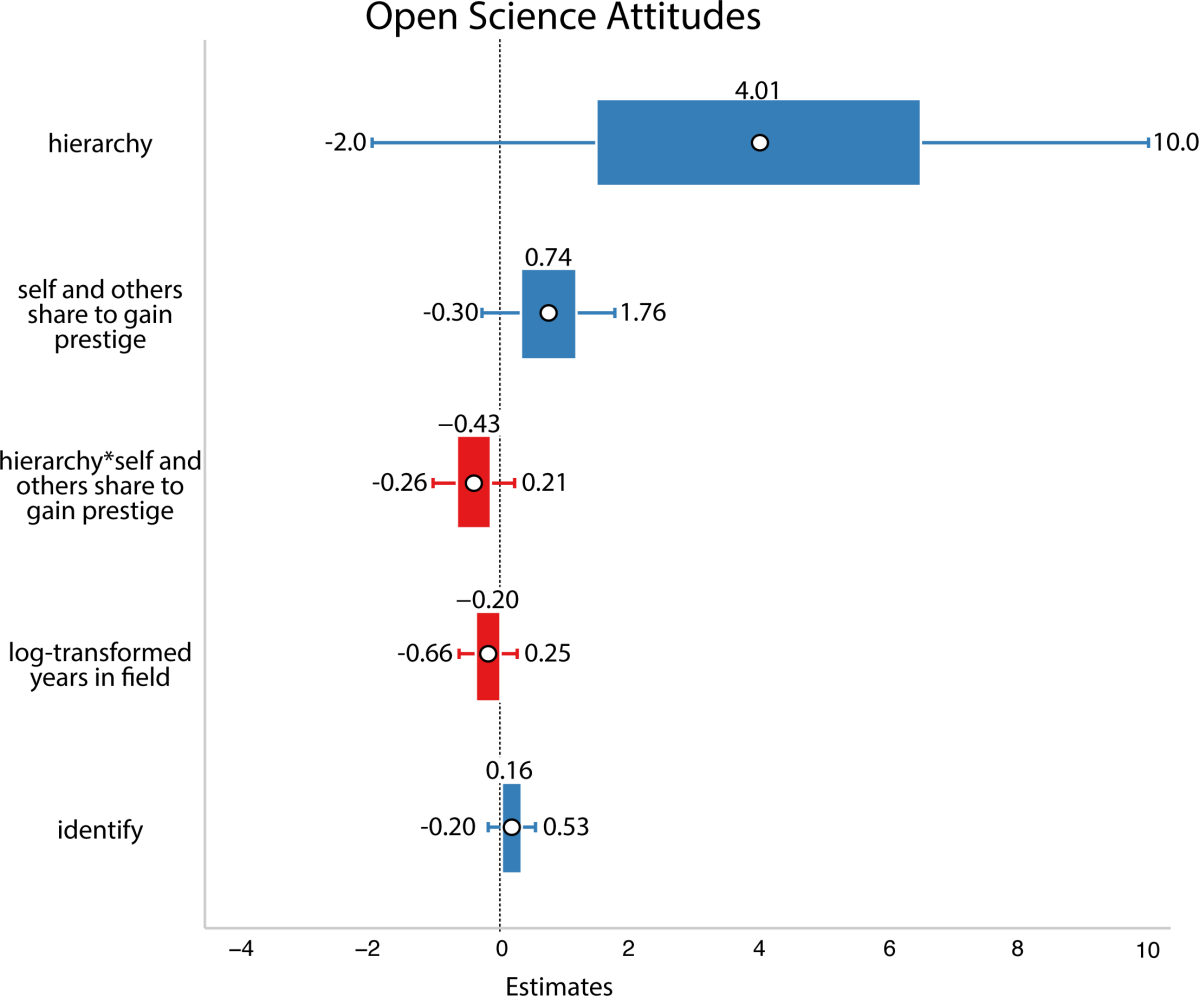
Results from Study 3. White circles are estimated means, inner bars are 50% highest density intervals and outer bars are 89% highest density intervals. Plotted using the R package sjPlot [33]. Here, we again found no evidence that people’s perceptions of the social dynamics in their field interacted with motivations to predict open science attitudes.

We did not find support for our hypotheses, there were no effects in the interactions or in the main effects (refer to figures 11 and 12).

Here, we tested a model that included interactions between the zero-sum/hierarchical factor and whether they, or others, shared to gain prestige, how much they identify with their field and how many years they have been in their field (log-transformed).

## 6. General discussion

In these studies, we tested the hypothesis that researchers’ perceptions of the social dynamics of their field predict their open science attitudes and plans. We operationalized open science attitudes as plans to publicly share manuscripts/preprints, code, stimuli/instruments and data, as well as participants’ perceptions of the importance of these practices. In Study 1, we first tested whether researchers have a coherent view of the social dynamics of their fields. Most previous work that asks how people perceive real-world social groups investigates stereotypes of large societal groups (e.g. the wealthy or immigrants) and has found that people see these groups along two dimensions: warmth and competence. However, in other types of social groups, specifically, those in which social coordination is necessary, such as teams, workplaces or academic fields, perceptions of the warmth and competence of individuals might be less important in decisions about how to interact with others. Instead, social dynamics, or rules of engagement, may better inform decisions about how to interact with others. We focused on two dimensions with which social groups can vary—how hierarchical/participatory a group is and how cooperative/competitive a group is—and investigated whether researchers recognize these dimensions, which would support the hypothesis that they are an important part of people’s social perception of this group. We found support for the prediction that researchers recognize the social dynamics of their field: we found evidence for one factor that included participants’ answers to eight questions about the social dynamics of their field. Importantly, participants’ answers about the characteristics (warmth and competence) of individuals in their field did not load onto this factor. This suggests that researchers infer the social dynamics of their field and see these dynamics as distinct from the characteristics of people in their field. We found similar results across Studies 2 and 3, which tested researchers from different disciplines: in Study 1, the participants were mostly social scientists, and in Studies 2 and 3, the researchers were mostly in the life and natural sciences. Thus, researchers across many scientific disciplines recognized social dynamics of their fields. However, while we hypothesized that these two dimensions (hierarchy/participatory decision-making and competition/cooperation) would be distinct from one another, the factor analyses did not support this hypothesis: we did not find clear evidence for two factors; rather, we consistently found evidence for one factor that captured 8 out of the 10 questions. It is possible that we did not ask the correct questions to capture these dimensions, or that these are not the most relevant dimensions the participants consider. Future work could test whether other questions better capture scientists’ perceptions of social dynamics and their role in generating scientific knowledge.

The central hypothesis of this article was tested in Studies 2 and 3. We consistently found that the more researchers cited ‘cooperating with other researchers’ as their motivation, the more they planned to share materials and manuscripts. However, across these two studies, our central hypothesis was not supported: the perception of social hierarchy/competition did not predict open science plans, either in Study 2 or 3. This was true when we tested whether the perceptions alone predicted open science plans, and when we tested whether they interacted with perceived and reported motivations to practice open science. Contrary to our hypothesis, it does not seem that perceived social dynamics in a discipline, the perceptions or reported motivations to practice open science, nor a fit between the two, are key predictors of open science attitudes. Additionally, we found no support for the concern that perceiving one’s discipline as characterized by hierarchy and competition would dampen intentions to engage in open science.

We also found a mismatch in people’s reported motivations to practice open science and their perceptions of other people’s motivations. In Study 2, in exploratory analyses, we found that people said that when they practised open science, they did so for prosocial reasons, like cooperating with other researchers or for the public good. However, the same participants said that when others shared, it was because of requirements to do so, or for personal benefits such as prestige. In Study 3, we replicated this finding in an independent sample of researchers with a very high rate of participation. These findings are consistent with self-enhancement biases, including the fundamental attribution error, and past evidence of mismatches between people’s reported open science practices and perceptions of other people’s open science practices (e.g. [38]). Thus, simply informing researchers that others are highly supportive of open science and reporting practising open science to cooperate with others and for the public good could increase the uptake of open science practices. This prediction could be tested in interventions.

**Figure 13.**
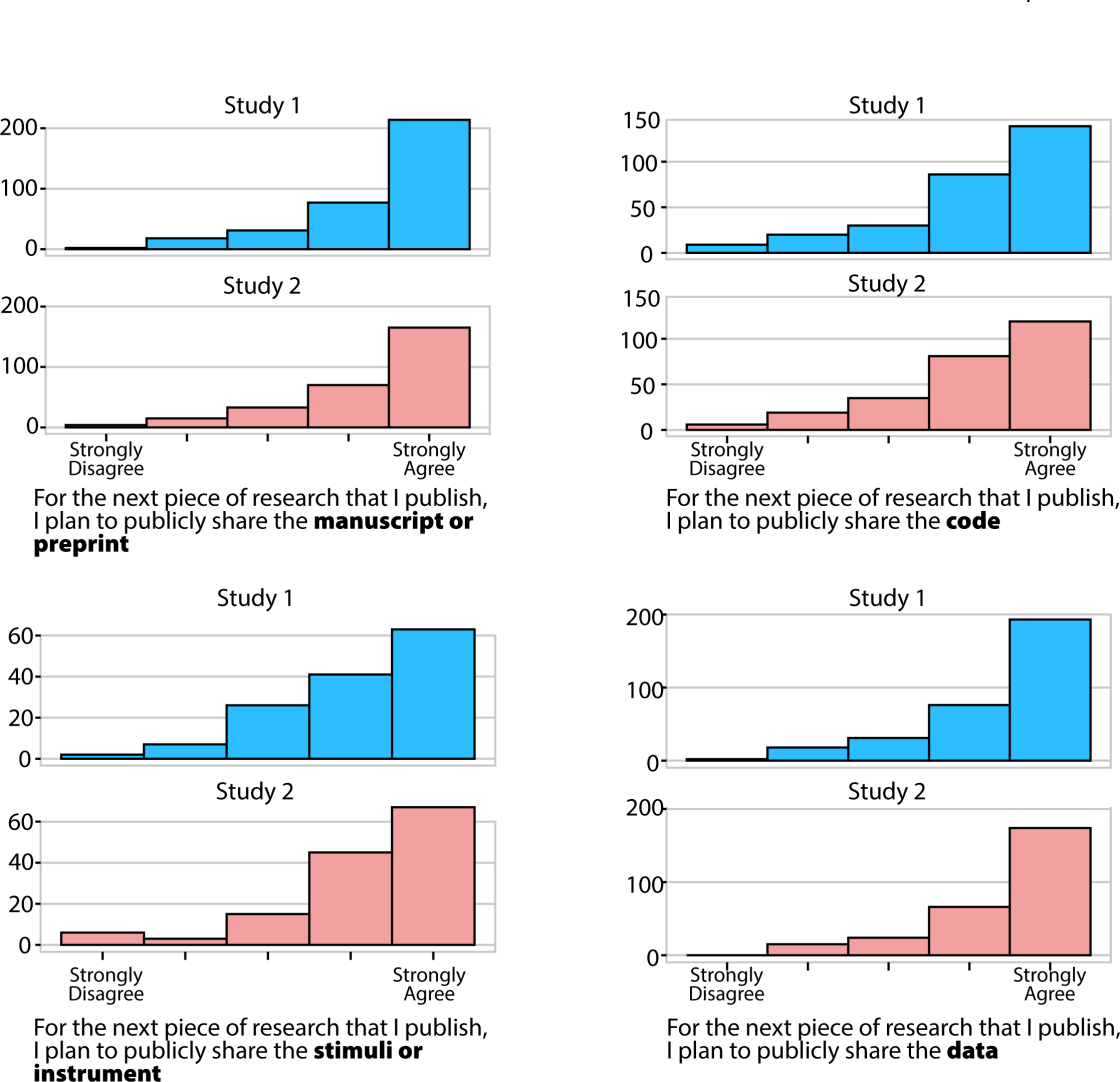
Histograms of answers to questions about plans to implement open science. *Y*-axes have different numbers because participants were not included if they said that the question did not apply to their field, or said they had no control over whether they could share.

Across two studies, researchers reported very high levels of support for open science (refer to figure 13). This was true in both the opt-in sample in Study 2 and the representative sample in Study 3 (refer to [37] for analyses). Before conducting the studies, we expected that there would be much more support for these open science practices in the sample in Study 2, compared to the sample in Study 3. While both studies were voluntary, the sample in Study 2 required that someone take time to read an email and opt into taking a survey that would be completed on their own time. In contrast, in Study 3, the time during which people took the survey was during an otherwise scheduled laboratory meeting, generating a very high participation rate for Study 3. The results suggest that the high levels of support for open science observed in both samples were not the result of biases that lead people who support open science to participate.

However, these high levels of support for open science may have impeded our efforts to measure what predicts them. It is possible that if we had a sample of researchers whose open science views had more variance, or had many more researchers complete our survey, we would have found support for our hypotheses. Moreover, it is possible that perceptions of social dynamics that we did not measure may correspond to people’s open science attitudes. For example, while we tested perceptions of hierarchy, we did not test whether people thought that this hierarchy was legitimate, merit-based and/or warranted (e.g. [39,40]). For example, if people see their field as hierarchical and illegitimate, they may be less motivated to cooperate with other researchers by practising open science. We also did not measure many other factors, such as whether trainees expect to stay in academia, how competitive they perceive the job market to be, where they have learned about open science practices, their interactions with open science advocates and their emotional, moral or practical framing of decisions about open science, etc. These could all lead to different levels of uptake in open science practices.

One limitation of this study is the extent to which the sample represents a larger population of researchers. The current results reflect open science attitudes at a research-focused institution in the United States, in five departments of a school of science. Open science attitudes may differ in other fields such as medicine, or fields that use qualitative methods. Other research has shown that attitudes toward open science vary by discipline (e.g [41–43]). The institution may have an especially established culture of open science practices, leading to high rates of support for them. This may have led to a lack of variance in open science attitudes, which may have contributed to our null findings. Another limitation to the interpretation of the findings was the set of open science practices we asked about. We chose practices that could apply to many fields and data sharing was not mandated in the United States in 2021 when we carried out this survey (the Nelson Memo was implemented soon in 2022). However, it is possible that if we had chosen to measure attitudes of less established and more controversial practices such as pre-registration, registered reports, open peer review, multi-laboratory replication projects or life cycle publishing, we would have found more variance in attitudes and correlations with perceptions of social dynamics. Finally, we did not measure people’s actual open science behaviour. It is possible that people overestimated their future open science behaviour. Future studies could investigate whether these factors predict people’s actual behaviour.

What practical implications do these findings have? While interpreting null results is difficult, one implication may be that people should focus more on infrastructure and systems that could make it easier for people to adopt and implement open science practices. This may be especially true given the high levels of support for open science we found across the two studies.

In conclusion, we found little evidence that people’s perception of the social dynamics, or rules of engagement, in their field, affected their open science practices. However, we did find evidence that people perceive a dimension of social dynamics in their academic fields that is distinct from individual traits, that people underestimate others’ cooperative motivations for engaging in open science and that, at least in this context, there are very high levels of support for open science. This suggests that many institutions could provide more support for open science that would be popular among their communities.

## Data Availability

All data will be on the OSF [44]. Supplementary material is available online [45].
